# Avoiding the Road to Nowhere: Policy Insights on Scaling up and Sustaining Digital Health

**DOI:** 10.1111/1758-5899.12909

**Published:** 2021-05-17

**Authors:** Amnesty LeFevre, Sara Chamberlain, Neha S. Singh, Kerry Scott, Purnima Menon, Peter Barron, Rajani R. Ved, Asha George

**Affiliations:** ^1^ University of Cape Town; ^2^ BBC Media Action India; ^3^ London School of Hygiene and Tropical Medicine; ^4^ Johns Hopkins Bloomberg School of Public Health; ^5^ International Food Policy Research Institute; ^6^ University of Witwatersrand; ^7^ National Health Systems Resource Center; ^8^ University of Western Cape

## Abstract

Digital health solutions offer tremendous potential to enhance the reach and quality of health services and population‐level outcomes in low‐ and middle‐income countries (LMICs). While the number of programs reaching scale increases yearly, the long‐term sustainability for most remains uncertain. In this article, as researchers and implementors, we draw on experiences of designing, implementing and evaluating digital health solutions at scale in Africa and Asia, and provide examples from India and South Africa to illustrate ten considerations to support scale and sustainability of digital health solutions in LMICs. Given the investments being made in digital health solutions and the urgent concurrent needs to strengthen health systems to ensure their responsiveness to marginalized populations in LMICs, we cannot afford to go down roads that ‘lead to nowhere’. These ten considerations focus on drivers of equity and innovation, the foundations for a digital health ecosystem, and the elements for systems integration. We urge technology enthusiasts to consider these issues before and during the roll‐out of large‐scale digital health initiatives to navigate the complexities of achieving scale and enabling sustainability.

## Background

The immense promise of technology to address critical impediments to accessing health information and services has given rise to the rapid proliferation of digital health solutions. A decade ago, the digital health landscape was littered with small scale deployments of digital health solutions with similar functionality, in overlapping geographies, often targeting the same cadres of health workers. This affliction of ‘pilotitis’ has largely waned, giving way instead to a number of high‐profile digital health solutions at scale involving large numbers of users. The most notable examples include the use of mobile phones for health information messaging and for health worker training, data capture and decision support tools. Despite the success of these programs in reaching scale, their sustainability remains uncertain.

**Figure 1:**
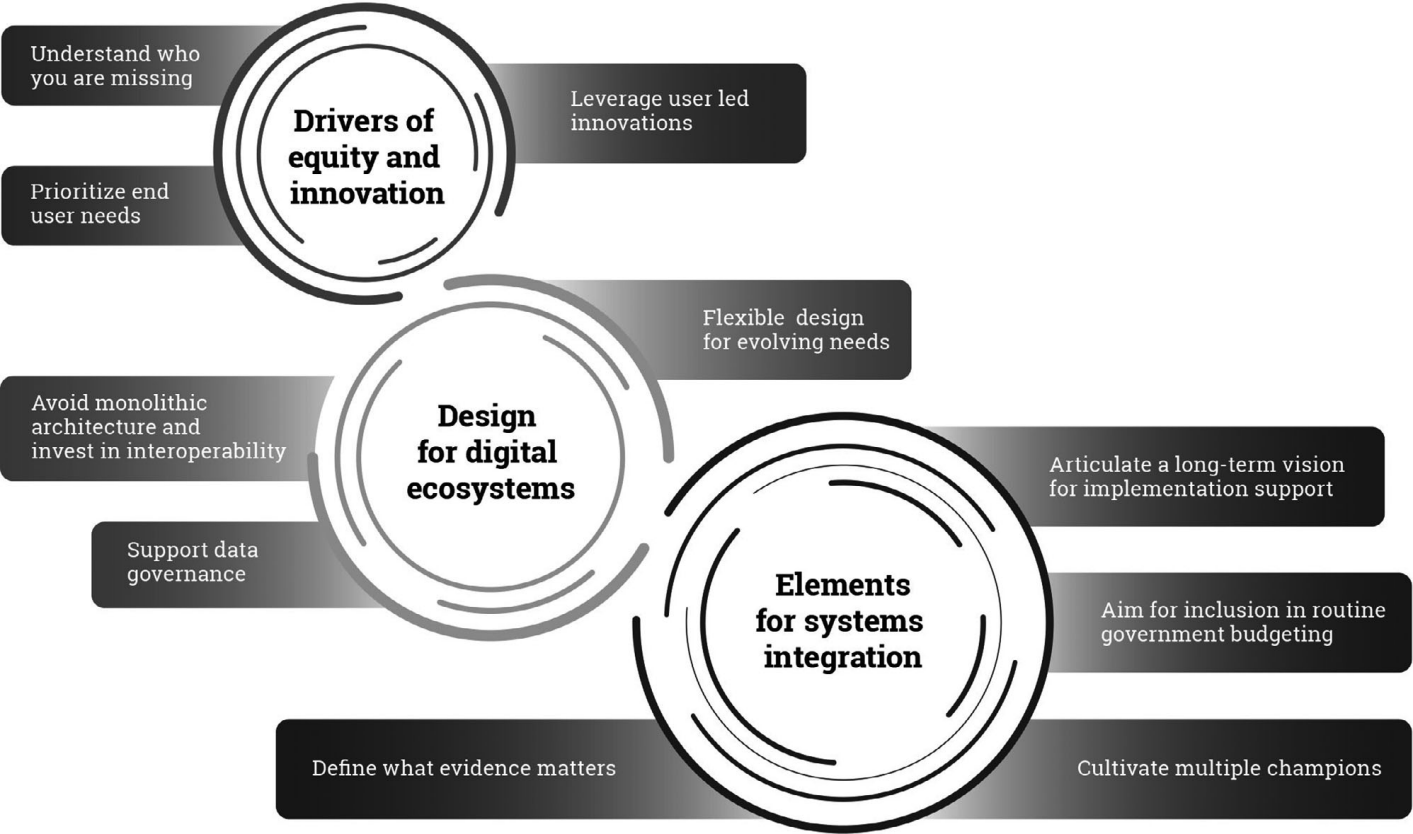
Conceptualizing policy considerations for scaling up and sustaining digital health solutions in LMICs

The Principles for Digital Development, launched in 2017, outline 9 items to consider in designing digital health programs to mitigate predictable and preventable factors contributing to program failure (Principles for Digital Development 2020). These items include design with the user, understand the existing ecosystem, design for scale, build for sustainability, be data driven, use open standards/ data/source/ innovation, reuse and improve, address privacy and security, and be collaborative. In this commentary, we have sought to be more specific than the broader principles and give a range of examples to provide a clearer path to action. As researchers and implementors, we draw on experiences of designing, implementing and evaluating digital health solutions at scale in number of settings across Asia and Africa, while providing examples from India and South Africa to illustrate ten considerations to support scale and sustainability of digital health solutions in LMICs. These can be categorised as: (1) drivers of equity and unforeseen innovation; (2) foundations for a digital health ecosystem; and (3) elements for systems integration as detailed in Figure 1.

## Drivers of equity and innovation

### Understand who you’re missing

Despite increasing access to mobile phones, social fault lines continue to inhibit ownership, access and use of digital technology. Women are 8 per cent and 28 per cent less likely than men to own a mobile phone across LMICs and in South Asia, respectively (Rowntree and Shanahan, 2020). Among mobile phone owners in LMICs, women use a smaller range of mobile services and are 20 per cent less likely than men to own a smart phone (Rowntree and Shanahan, 2020). Limitations in women’s numeracy and literacy along with restrictions in women’s autonomy and agency further impede their access to, and use of, mobile phones. In many contexts, migration for employment or relocation due to pregnancy and childbirth further impacts women’s access to mobile phones. In Madhya Pradesh, India, cross‐sectional surveys of pregnant women to assess digital competency found that only 85 per cent of women with access to a phone could dial a phone number and only 38 per cent could open and read a text message (LeFevre, 2020). When interviewed following childbirth at 12 months postpartum, only 44 per cent of this same population of women had the same phone number – a indication that SIM change remains a significant impediment to continuous engagement over time (LeFevre, 2020). Women’s phone use was also limited by data inaccessibility in terms of affordability, along with social norms that discouraged women from visiting the male‐dominated shops for phone top‐ups. Consideration of the lived realities of marginalized populations where gender intersects with multiple other social determinants and how that shapes access to, and use of, mobile phones over time is integral to understanding not only who a digital health program can reach, and to what degree, but most importantly who it risks excluding. People at the lowest end of the socio‐economic spectrum, particularly women, are less likely to own or have access to a cell phone and less likely to have access to internet and data.

### Identify all user needs but prioritize end‐users’ needs

While adherence to the Principles for Digital Development mentioned earlier should result in the identification of technology needs by users of the digital solution across the health system, digital health solutions cannot be everything to everyone and some degree of prioritization is inevitably required. Three questions should be considered in weighing the needs of different users: Which features will have a direct impact on population health? How feasible will the implementation of these features be? How will they benefit the end users of the digital solution?

One often touted benefit of data capture applications for health workers is that they streamline cumbersome paper‐based registers consolidating multiple records onto a single tablet or mobile device. Less discussed, however, is whether all of those data elements are needed in the first place and whether in turn it makes sense to digitize all of them. The capture of these data can be time‐consuming, detract from vital time providing patient care, and be of little benefit to the patient or provider (who often merely pushes the data upstream). In this example, the need of health workers for only essential data to be captured should be prioritized over the needs of more powerful policy makers who often want more data collected due to potential accountability questions, but that are not used for routine decision‐making.

### Leverage user led innovations

Innovation may occur in unanticipated and unintentional ways which can provide added value. For the Integrated Child Development Services‐Common Application Software (ICDS‐CAS) data capture and decision support solution in India, health workers were provided with a mobile handset, the app, data, and unrestricted downloads of other applications onto the handset. The latter enabled health workers to download WhatsApp and use it along with voice calls in parallel with CAS, to communicate with supervisors, other health workers, and their beneficiaries to provide enhanced services (Gopalakrishnan, 2020). In the wake of COVID‐19, this has provided a vital lifeline to continue service provision to mothers and children where face‐to‐face services are contra‐indicated (Nair, 2020). In some respects, the handset, data, and access to WhatsApp have become as important to continuous, quality nutrition and counseling services as the CAS application itself.

## Foundations for digital health ecosystem

### Design solutions for evolving needs

To enable sustained use, digital health solutions should be flexible and adaptable, balancing configurability (e.g. accommodating plugins to modular software frameworks) and customizability (e.g. changes to underlying software frameworks) to address evolving health systems and program requirements. In India, Mobile Academy, the interactive voice response‐based training course for health workers on reproductive maternal newborn and child health, had been redesigned for the Department of Drinking Water and Sanitation to create a training platform capable of running multiple courses of configurable length at the same time. New curriculums and courses can be launched with minimal added cost. The non‐communicable diseases (NCD) app has similarly been configured to include a cervical cancer screening module; a feature which can be turned‐off or on depending on local needs. To sustain use by health workers over the long term, it is vital that digital health solutions are designed to accommodate evolving changes to expanding service delivery provision and needs.

### Avoid monolithic architecture and invest in interoperability

Few countries globally have a national digital health architecture that enables diverse applications to easily utilize common resources such as national data registries. Instead most countries have siloed, standalone applications or in some instances, data exchange between two or more applications (WHO, 2020). Gaps in government capacity and resources, coupled with the political challenges of obtaining consensus across stakeholder groups (including different ministries; federal and state authorities), have meant that establishing a fully exchanged digital health system is unlikely in the short term in most settings. The result is often an immediate and important focus on integrating applications with routine health information systems (e.g. District Health Information systems‐2 (DHIS2) or the mother and child tracking system (MCTS) in India). The MomConnect program’s integration with DHIS2 in South Africa provides the denominator for assessing coverage of women attending antenatal clinics who receive messages. Despite the advantages of linkages with routine health information systems, these alone are likely to be inadequate for two reasons: (1) the proliferation of applications capturing data in the same geographies necessitates data exchange to avoid duplication and improve health services delivery; and (2) the quality of routine health information systems data are often poor. The latter can limit the coverage of programs which draw upon these data for their subscriber base. To avoid the road to nowhere, investment, government leadership and oversight is further required to design national health information system architecture that is modular, and based on agreed data exchange standards for structural interoperability (including unique patient identifiers), medical coding standards for semantic interoperability, and documentation processes for foundational interoperability (NHSRC‐India, 2020).

### Support data governance even though no‐one’s watching

Standards for digital health data governance are emerging and include standards for ethics and informed consent, data access (procedural oversight and structural controls), as well as legal and regulatory policies (NHSRC‐India, 2020; Tiffin et al., 2019). In the European Union, the General Data Protection Regulation (GDPR) outlines data protection principles, rights and obligations enforced by the Information Commissioner's Office (ICO, 2020); arguably serving as the global gold standard for personal protection of information. The South African government's Protection of Personal Information Act (enacted in 2014, commenced July 1 2020) provides a similar standard of data governance protections to personal data privacy. In India, while legislation has been proposed, it has yet to be enacted. Understanding that the future must favor protection of patient rights and privacy, digital systems should be designed in accordance with best practice from the outset even if legislation has not been enacted and regulatory bodies have yet to exist to enforce them. This is critical for avoiding costly retrofitting, protecting the rights and privacy of individuals, and ensuring that valuable data are not mined by organizations that have unauthorized access.

## Elements for systems integration

### Articulate a long‐term vision for implementation support

From the outset a long‐term vision for implementation support needs to be defined inclusive of specified roles and responsibilities over time for key stakeholders. The capabilities and resources required to strategically manage, maintain and support digital solutions after scale up should be considered along with human resource requirements, infrastructure and connectivity requirements, application maintenance to ensure security over time, the cost of complying with potential changes in government regulations.

The process for defining this long‐term vision should aim to recognize that external support from donors is seldom indefinite, and the resources required along with the capacity of key stakeholders may need to be redefined and evolve over time.

### Inclusion in routine government budgeting is essential, but has trade offs

Designated government funding is required to sustain the implementation of digital health solutions in the public sector. However, the pathway for securing it may not always be straightforward or the amount sufficient. Further restrictions on the use of government money may impede implementation by impacting procurement, including the procurement of third‐party software and hardware and ongoing maintenance and support from vendors with the necessary skillsets. Continued donor funding can be difficult to sustain but nevertheless provides added flexibility to be responsive to evolving needs on the ground. For full‐scale sustainability, financing for all aspects of digital health solutions will ultimately need to be integrated into routine health budgets and budgeting processes. This entails not just costing and budgeting for the long term but building and sustaining relationships that navigate the corridors of power between different stakeholders across government and funding agencies.

### Define what evidence matters

The perceived trajectory of a successful public health intervention is often through a pathway of demonstrating efficacy and then effectiveness prior to scale. The technology space has deviated from this model to focus instead on iteration and learning‐while‐doing. For digital health solutions which emphasize administrative data capture needs, and are not directly linked to service delivery, assessing linkages with health outcomes is not always necessary. However, in some settings, robust impact level evaluations of public health outcomes will be required to justify government resources. In South Africa, the MomConnect program focused its evidence gathering primarily on implementation research required to monitor population coverage, reach, and system feedback, as well as user perceptions of the health information messages. Six years later, the program continues to enjoy high coverage across all provinces along with government support. However, it has not been successfully mainstreamed into routine public health sector operations and continues as a kind of ‘add‐on’ service. Had sufficient investment been made in research on population health impact and cost been invested in, the case for its integration into routine healthcare delivery would have been difficult to ignore.

### People matter even though they move on

Digital health solutions require one or more ‘champions’ to scale, including buy‐in from the highest levels of government and donors with significant resources. In India, for ICDS‐CAS, high level government support was frequently cited as being integral to the program’s successful scale up. In South Africa, the idea for MomConnect came from the former Minister of Health based on a similar pilot initiative he saw on a field visit. His engagement with the program, including ‘road‐shows’ undertaken to provinces to support its rollout, is widely credited with its timely and successful scale up. For both ICDS‐CAS and MomConnect, these original champions so integral to the programs’ successful scale‐up have since been transferred or moved on to other positions. While the long‐term stability of both programs remains uncertain, their implementation continues. To be sustained, the ownership of these solutions must be agnostic to individual persons or organizations, and their value to the health system continuously renegotiated as part of ongoing relationship building.

## Conclusions

Digital health solutions offer tremendous potential to enhance the reach and quality of health services and population‐level outcomes in LMICs. While the number of programs reaching scale increases yearly and planning guides are being developed (WHO, JHU‐GMI, and UNF, 2015), the long‐term sustainability for most remains uncertain; many have struggled to become fully integrated components of government health systems. Given the investments being made in digital health solutions and the urgent concurrent needs to strengthen health systems to ensure their responsiveness to marginalized populations in LMICs, we cannot afford to go down roads that lead to nowhere. These 10 considerations related to the drivers of equity and innovation, the foundations a digital ecosystem, and the elements for systems integration highlight the complexities of navigating scale and ensuring sustainability. Technology enthusiasts must consider these issues before and during the roll‐out of large‐scale digital health initiatives.

This work was support by Countdown 2030 with funding from BMGF (INV‐007594 / OPP1148933). It is also supported by the South African Research Chair’s Initiative of the Department of Science and Technology and National Research Foundation of South Africa (Grant No 82769) and the South African Medical Research Council. Any opinion, finding and conclusion or recommendation expressed in this material is that of the author and none of the funders accept any liability in this regard.
